# Frailty Triage: Is Rationing Intensive Medical Treatment on the Grounds of Frailty Ethical?

**DOI:** 10.1080/15265161.2020.1851809

**Published:** 2020-12-08

**Authors:** Dominic J. C. Wilkinson

**Affiliations:** a University of Oxford; b John Radcliffe Hospital; c Murdoch Children’s Research Institute

**Keywords:** Age, discrimination, frailty, intensive care, rationing

## Abstract

In early 2020, a number of countries developed and published intensive care triage guidelines for the pandemic. Several of those guidelines, especially in the UK, encouraged the explicit assessment of clinical frailty as part of triage. Frailty is relevant to resource allocation in at least three separate ways, through its impact on probability of survival, longevity and quality of life (though not a fourth—length of intensive care stay). I review and reject claims that frailty-based triage would represent unjust discrimination on the grounds of age or disability. I outline three important steps to improve the ethical incorporation of frailty into triage. Triage criteria (ie frailty) should be assessed consistently in all patients referred to the intensive care unit. Guidelines must make explicit the ethical basis for the triage decision. This can then be applied, using the concept of triage equivalence, to other (non-frail) patients referred to intensive care.

## INTRODUCTION

Critical care is a limited healthcare resource. In many countries, adult Intensive Care Units (ICU) often operate at close to capacity and must decline a number of patients referred for possible admission (Rhodes et al. 2012). Some patients are judged to be too well to benefit from intensive care, while others are thought to have too poor a prognosis (Joynt et al. 2001). Triage for ICU admission is ethically complex and has been reported to vary between hospitals and clinicians (Evans et al. 2011; Wilkinson and Truog 2013).

**Figure 1.**
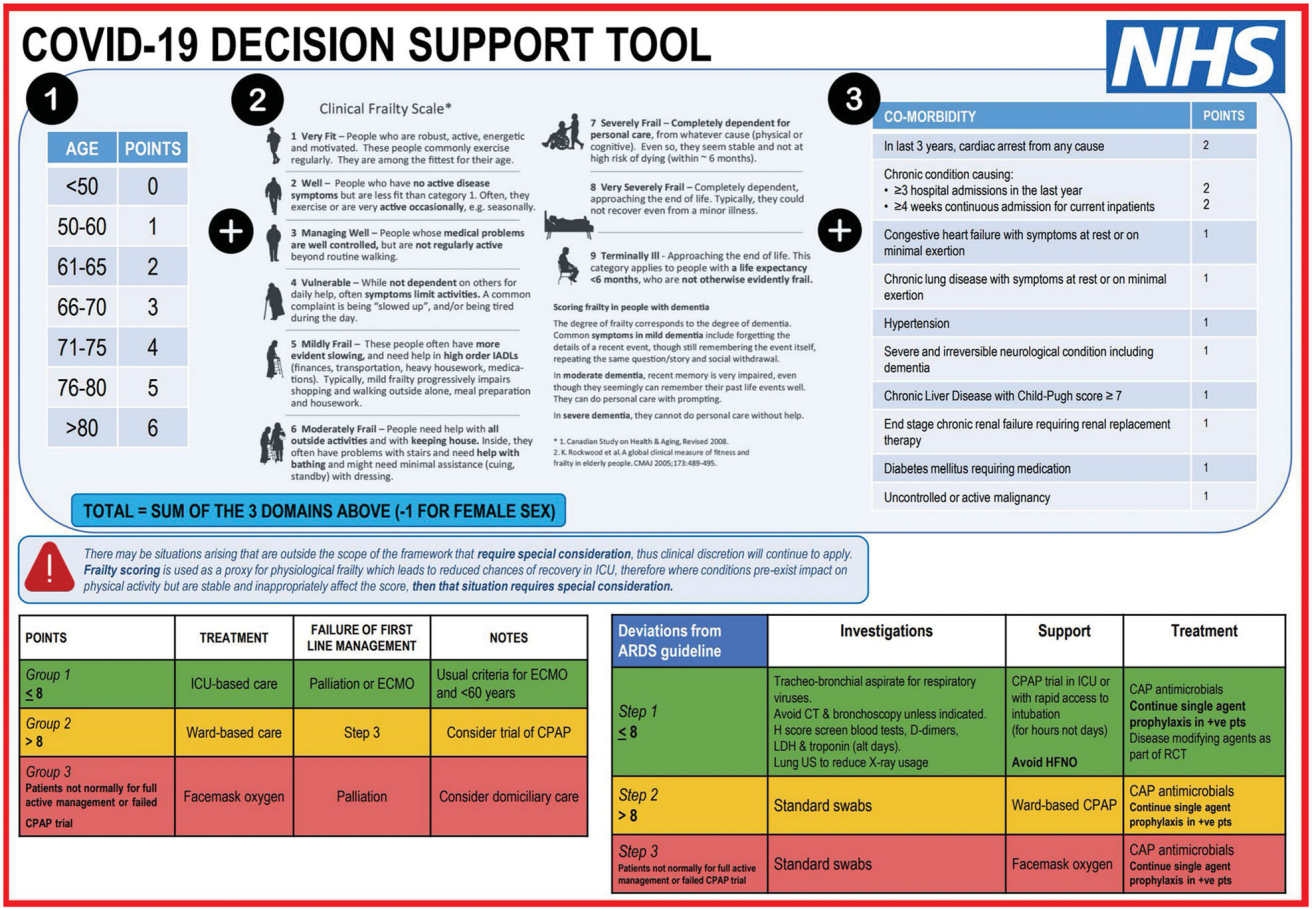
Draft COVID-19 Decision-support tool (not implemented) (@pmdfoster 2020). Permission to reproduce granted by Peter Foster June 18, 2020.

In early 2020, the need for ICU triage reached wide public attention. Health professionals sought to develop guidelines for critical care in the context of the pandemic. This concern was motivated by reports of the experience from Northern Italy, where hospitals had been overwhelmed by the numbers of patients with severe COVID-19 (Bauchner 2020; Grasselli et al. 2020; Rosenbaum 2020). Initial Italian approaches to triage involved admitting patients on a first-come-first served basis. However, this meant that patients arriving later were potentially unable to access intensive care (Craxì et al. 2020). There were reports that some Italian hospitals were using age cutoffs for access to intensive care (Lintern 2020). Pandemic specific guidelines were produced to try to ensure a consistent ethical approach. Countries varied in which factors they emphasized for decision-making (Antommaria et al. 2020; Joebges and Biller-Andorno 2020). In the UK, several guidelines promoted the assessment of clinical frailty as a tool to inform intensive care decisions (see Figure 1). One potential justification was that such an approach might avoid age-based discrimination (Wysong 2020). It is unclear to what extent frailty was used in decision-making about provision of intensive care in UK intensive care units. The numbers of critically ill patients in the UK did not end up being as great as initially feared. However, anecdotally, some UK patients died after being denied intensive care admission, possibly on the grounds of their age and perceived frailty e.g. see (McConville 2020; Spackman 2020). Frailty was also included (as one factor among several to be considered) in guidelines from France, Belgium and Ontario, Canada (Azoulay et al. 2020; Downar 2020; Meyfroidt et al. 2020).

Given the possibility of subsequent further surges of intensive care demand (particularly over winter), it would be important to ethically evaluate the use of frailty in intensive care triage. I will start by outlining the concept of frailty and its relevance to outcome in intensive care. I will then analyze the potential ethical role of frailty in decisions, drawing on the example of UK guidelines. I address concerns, including that this would constitute problematic discrimination against the disabled or elderly or reinforce social inequality. It is beyond the scope of this paper to review in detail other possible approaches to ICU triage, or other possible criteria to include in allocation. Nevertheless, in the final part of the paper, I will consider the potential for including frailty in US triage guidelines, and the merits of an approach taken in Ontario, Canada.

## FRAILTY

The word “frail” has the same etymological source as “fragile,” and indeed in general use has similar connotations—referring to a state of being easily damaged, broken or destroyed (Oxford English Dictionary 2020). When used in relation to people, this is often linked with advanced age. The concept of frailty has long been recognized within geriatric medicine as a useful way of capturing the variable vulnerability of older patients to illness (Vaupel et al. 1979). Patients with similar age can differ widely in how likely they are to become seriously unwell or die (for example from pneumonia or following a fall). “Frailty” is not, itself, a distinct disease or illness; rather, it represents a cluster of features that commonly co-occur, particularly toward the latter phase of life (Ranzani et al. 2020). Frailty exists on a spectrum, from patients who have some mild features, to those who are extremely frail. It is typically progressive (Harrison et al. 2015).

**Figure 2.**
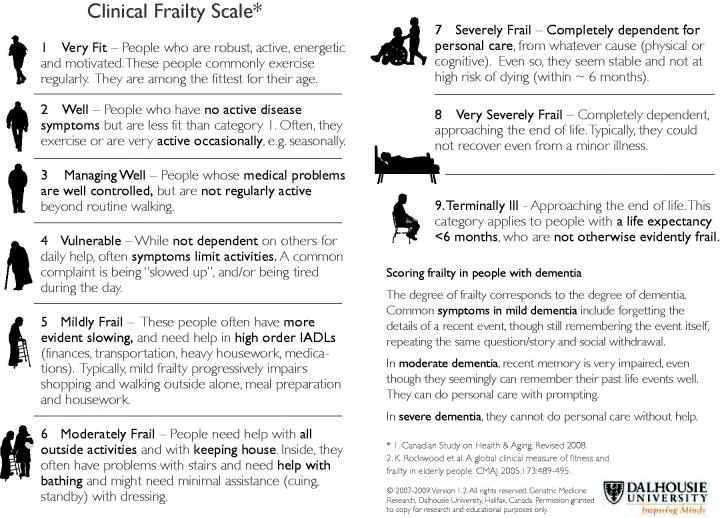
Clinical Frailty Scale. Reproduced with permission K Rockwood.

The essential underlying feature of the state of frailty is the loss of biological reserve combined with failure of homeostatic mechanisms (Flaatten and Clegg 2018). These, in turn, generate the state of vulnerability or fragility that makes adverse outcomes more likely (ie falls, disability, cognitive decline, hospitalization and need for care). Like other clinical syndromes, frailty is often diagnosed not on the basis of a particular test result—rather by the presence of a combination of features. These can be assessed as part of a comprehensive geriatric evaluation (Turner and Clegg 2014); however, this is time-consuming to perform properly and impossible for a patient who is acutely unwell. A number of clinical tools have been developed to allow rapid evaluations. These are sometimes based on the frailty phenotype—a combination of several of reduced strength, slowing, tiredness, loss of weight, and reduced activity levels (Lacas and Rockwood 2012). Others evaluate frailty on the basis of co-morbidities. For example, a “modified frailty index” generates a score based on how many of a set of illnesses are present eg congestive cardiac failure, hypertension, strokes, myocardial infarction, etc (Flaatten and Clegg 2018). One tool based on an overall clinical judgment, the “Clinical Frailty Scale” (CFS) has been widely used because of its ease of use, and ability to be assessed even during an acute illness, or based on history from family members (Pugh et al. 2018). The original CFS was a seven-point scale, and was found to be highly correlated with more complicated scoring systems and with outcome over a 5 year period (Rockwood et al. 2005). The current form of the CFS includes a simple visual scale from 1 to 9 and is based on levels of activity as well as the support needed for daily activities (Figure 2). Level 9 represents patients with terminal illness and short life expectancy who are not otherwise frail. The level of frailty is also equated with the severity of dementia.^1^

## FRAILTY AND OUTCOME

Frailty is associated with life expectancy. In Rockwood’s original description of a community cohort of older Canadians (age 65 and older), each higher category of frailty was associated with about a 20% increase in the risk of death over a 5 year period (Rockwood et al. 2005). Patients with a frailty score of 7 had a median survival of about 40 months. Frailty has been particularly linked with survival in patients who require hospital admission. A 2017 meta-analysis found that frailty was associated with higher in-hospital and long-term mortality (Muscedere et al. 2017). In the “VIP1” (Very old Intensive Care Patients) study, 5,021 patients aged 80 or over were followed up after admission to intensive care units in 21 European countries (Flaatten et al. 2017). There was a linear relationship between the clinical frailty score and mortality. The authors observed about a 30% 30-day mortality for patients with a CFS of 3 requiring emergency intensive care admission, rising to 75% mortality for CFS 9. However, the absolute rate of mortality in older frail patients admitted to intensive care varies between studies eg see (Darvall et al. 2019; Muessig et al. 2018).

While many of the studies examining the relationship between frailty and outcome have focused on older hospitalized patients, frailty also impacts survival in younger patients. In a very large cohort from a UK biobank (almost 500,000 participants), frailty and pre-frailty was associated with increased mortality over a median of 7 years of follow-up in younger as well as older adults (with the exception of women aged 37–45) (Hanlon et al. 2018). In a US intensive care cohort, frailty in patients younger than 65 was associated with lower survival (Brummel et al. 2017). Half of all patients with CFS > =5 were <65. Similarly, in a Canadian cohort of about 200 patients aged 50–65, frailty was linked with increased mortality risk over ensuing 12 months in adjusted analysis (Bagshaw et al. 2016). They concluded that the probability of survival is higher for younger compared with older frail patients, but their risk is age-shifted compared to peers—ie they have a mortality risk equivalent to an older chronological age.

Finally, frailty is also associated with disability and quality of life following intensive care admission. In one study, higher pre-admission CFS was linked with a higher probability of new or increased disability in instrumental activities of daily living at 3 months (Brummel et al. 2017). A meta-analysis found only a small number of studies reporting formal measures of quality of life, but two reported reduced health related quality of life at 12 months following ICU admission (relating to physical function) in frail patients (Muscedere et al. 2017). In a Canadian study, of patients >80 years who were moderately to severely frail prior to intensive care admission and who survived, only one quarter subsequently returned to their home over the ensuing year (Heyland et al. 2015).

## FRAILTY AND INTENSIVE CARE DECISION-MAKING

Frailty could be used in decisions about provision of intensive care in several different ways.

### Shared Decision-Making

One obvious and likely uncontroversial way that frailty might impact decisions is through its impact on patient preferences for treatment and shared decision-making (McDermid and Bagshaw 2009). Patients (and surrogates) should be informed about the patient’s chance of survival given their preexisting frailty as well as their likely long-term function and chance of returning to their home. This information would obviously be relevant to an informed decision, as well as to consideration of the patient’s wishes (in a situation where the patient lacked capacity to decide). As long as the information provided to the patient is relevant to their situation and accurate, there cannot be any major ethical objection to incorporating frailty into decisions in this way.^2^

### Futility

A different use of frailty scores might be through the identification of a threshold level of frailty such that intensive care admission would be futile—and thus may be declined.

There is extensive literature on the difficulty of identifying and defining futility in intensive care, as well as in using futility as a basis for declining intensive care admission (Truog et al. 1992; White and Pope 2016; Wilkinson and Savulescu 2011). Whether treatment is regarded as futile will clearly depend on the goal of treatment, what is regarded as an acceptable outcome, and what chance of that outcome is too low for treatment to be pursued. However, specifically in relation to frailty, it appears hard to define a level where treatment would be *physiologically* futile (ie that there is a zero or close to zero chance of achieving a physiological goal) (Bosslet et al. 2015). For example, in the VIP1 study, there were more than 100 emergency intensive care admissions of patients over 80 years assessed as “very severely frail” (with an expected life expectancy of <6 months, CFS 8). Those patients had a high mortality rate, but 40% survived for at least 30 days (Flaatten et al. 2017). If survival is possible after intensive care even for patients with the highest degree of frailty, it is difficult to see how frailty assessment could be used to identify when intensive care is physiologically futile.

Alternatively, frailty might be used to identify treatment that would be futile or “potentially inappropriate” on *qualitative grounds*—i.e., based on a judgment that the patient’s quality of life is sufficiently low that intensive care should not be provided even if desired by the patient/surrogate.(White and Pope 2016) However, there is considerable ethical difficulty in determining when treatment is qualitatively futile, and this concept has been heavily criticized (Truog et al. 1992; White and Pope 2016). Proposed definitions of qualitative futility (for example that the patient is permanently unconscious or permanently dependent on intensive medical care (Schneiderman et al. 1990)) would not apply to many frail patients, even those with the highest scores.

### Rationing

While treatment might not be strictly “futile” for patients with a high frailty score, it might still be ethical to withhold treatment. The justification would be on the basis that this would not be an appropriate use of limited healthcare resources (Wilkinson et al. 2018). It was clear that the UK NICE guidance (see below) was motivated by the anticipated high demand for intensive care in the context of the COVID-19 pandemic. Some of the key research on frailty and outcomes in intensive care was also apparently stimulated by concern for limited resources. McDermid and Bagshaw, writing in 2009, describe a “crisis” caused by insufficient availability of critical care and a conflict between the expectations of individuals, and the amount of resource that society/governments are prepared to make available. They specifically moot that evidence of frailty’s relationship with outcome could be used “to guide ICU triage and decision-making at a health policy/societal level” (McDermid and Bagshaw 2009). Kenneth Rockwood (the Canadian geriatrician who developed the clinical frailty scale), suggested in an interview in June 2020 that the use of this scale could prevent intensive care units being overwhelmed as had occurred in Italy (Wysong 2020).

## FRAILTY AND INTENSIVE CARE DECISION-MAKING IN THE UK

The National Institute for Health and Care Excellence (NICE) published a rapid clinical guideline on critical care for adults in the context of COVID-19 on 20th March, 2020. It was developed explicitly to support clinical decision-making in the setting of intense demand (@NICEComms 2020). The only specific factor mentioned in the guideline was clinical frailty. The first point of the NICE guideline recommended assessing all patients for their degree of frailty on admission to hospital (irrespective of their COVID-19 status). For patients potentially needing critical care with a clinical frailty scale (CFS) of five or more, the guideline recommended a discussion with critical care specialists about whether treatment would be appropriate.

The NICE guidance did not exclude patients with a CFS above 4 from critical care, though it appeared to imply a potentially more restrictive approach at this level or higher. For patients with a CFS <5, the algorithm indicated that they should be automatically referred to critical care (assuming that the patient wished for treatment).

The initial guidance implied that the CFS could be applied to all patients. On 25th March, (following criticism and a threatened judicial review) (Hodge Jones and Allen Solicitors 2020) NICE published additional clarification, indicating that the scale should only be calculated for patients over the age of 65. For patients younger than 65, including patients with stable learning disability, autism or cerebral palsy, the guidance recommended an “individualized assessment” of frailty (NICE 2020).

A draft UK national pandemic allocation guideline, developed in late March, proposed a scoring system for intensive care triage (Figure 1) (Foster, Staton, and Rovnick 2020). That scoring system incorporated age (score from 0 to 6 based on age bands <50, 50–60, 61–65, 66–70, 71–75, 76–80, >80), frailty (score 1–9) and co-morbidities (various illnesses given scores). Female patients had one point deducted from their score. It indicated that patients with a score greater than 8 would not be managed with invasive ventilation. This would have automatically excluded male patients aged 70 or higher with a frailty score of 5 or more, or female patients above 75 with a frailty score ≥ =5 (younger or less frail if they had comorbidities).

This guideline was reported to have been rejected by UK health officials (Kirkpatrick and Mueller 2020), and no official NHS guidance was produced.^3^ The Intensive Care Society—a national professional group of intensive care doctors, later published a version of the same basic approach (Intensive Care Society 2020). It recommended consideration of the same factors (age, frailty, comorbidity) but did not recommend a scoring system, nor any particular threshold for decisions, even in situations when there was a national shortage of ventilators and overwhelming demand. (The Intensive Care Society Guidance also did not recommend any difference in approach between male and female patients with COVID-19. It is beyond the scope of this paper to explore whether it is justified to include sex in triage decision-making. However, the arguments below in relation to age and disability potentially also apply to sex.)

## FRAILTY AND RATIONING

What would be the ethical justification for using frailty in resource allocation? As noted above, frailty is associated with several elements of prognosis that might be relevant to the allocation decision. I will first consider the arguments in favor of including frailty in allocation. I will then consider potential concerns, including that this would constitute problematic discrimination.

### Probability of Survival

**Figure 3.**
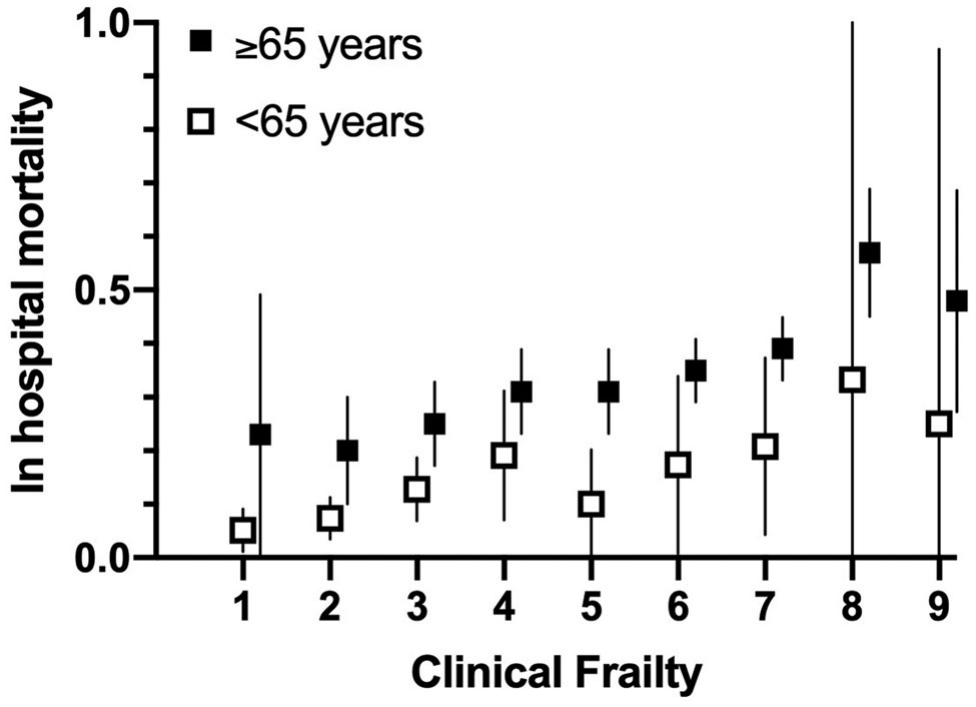
Short term mortality for inpatients admitted to 11 hospitals (UK, Italy) with COVID-19 in March/April 2020. Data extracted from Hewitt et al. (2020, Appendix 1). Mortality plotted with calculated 95% confidence interval. NB These represent mortality for all admissions and therefore are likely to be significantly lower than mortality for patients admitted to or referred to intensive care. For example, the average in-hospital mortality for patients admitted to critical care with COVID-19 in the UK has been reported to be 41%, rising to 50% in patients with any severe comorbidity (Intensive Care National Audit and Research Centre 2020). Lower mortality (and wide confidence intervals) for patients with the highest degrees of frailty (e.g., CFS 9) potentially reflects selective management of such patients at home and avoidance of hospital admission.

Firstly, frailty is linked to the chance of survival. Figure 3 illustrates recently published evidence of the relationship between clinical frailty scores and mortality for hospitalized patients with COVID-19 (Hewitt et al. 2020). Almost all international allocation guidelines relating to intensive care explicitly accept that where resources are limited it is ethical to preferentially allocate treatment to those patients who have the highest chance of survival (Wysong 2020). Twenty five out of twenty-six US ventilator triage policies endorsed this principle (Antommaria et al. 2020). Prioritization on the basis of probability of survival has been argued to be a universal allocation principle for life-sustaining treatment in the context of limited resources (Savulescu et al. 2020b). Such a principle means that more lives are saved. While some philosophers have defended equal treatment even where that would mean saving fewer lives (for example, tossing a coin to decide whether to send a lifeboat to save five people or one person) (Taurek 1977), other philosophers have persuasively argued that equal consideration of interests supports saving a greater number of lives (Parfit 1978). Surveys of the general public suggest that they overwhelmingly support this principle (Arora et al. 2016).

A standard way of incorporating probability of survival into ICU triage has been through assessment of illness severity (Antommaria et al. 2020). For example, a high severity of illness score (i.e. SOFA >11) has been associated with a predicted chance of mortality of 90%. Previous UK pandemic guidance (from 2009) recommended non-admission of patients with this level of severity of illness (Department of Health 2009). Guidance produced in Pittsburgh prior to the COVID-19 pandemic, but adopted by a number of states during the pandemic, assigned patients with this illness severity the lowest priority (though other criteria were also considered, see below) (White 2020; White and Lo 2020).^4^ One challenge would be in identifying a level of frailty which corresponded with a similar mortality. As already noted, data on frail patients admitted to intensive care suggests that even the most frail category is not necessarily associated with 90% mortality. Patients with a CFS of 5 or 6 would potentially have a chance of survival significantly higher than 10%. (One difficulty is that the cohort of patients with a particular frailty score who were admitted to intensive care is likely to be different from the wider group of patients with that level of frailty who might have become critically ill but were not admitted. Selection bias means that it is hard to determine the mortality (if intensive care were provided) for a group of frail patients who do not always receive intensive care admission.)

### Predicted Duration of Survival (Future Life Years)

Second, frailty is associated with duration of survival. It is more controversial, yet arguably clearly ethical, to give priority to patients who will survive for a longer period of time following intensive care admission (White and Lo 2020). In a setting where many patients are in need of respiratory support, it is relevant that someone might survive (after COVID-19) for 40 years, rather than die within a matter of months even if they recover from their acute illness. The principle of prioritizing patients who would survive longer has broad intuitive appeal (Arora et al. 2016). It reflects the widely accepted notion that life is valuable not merely in and of itself, but also because of the wellbeing that it brings. As individuals, we are not agnostic about choosing between treatments, one of which might prolong life for a short period, and one of which would offer us many more years of life. Similarly, when it comes to funding or prioritizing different treatments (e.g., cancer drugs), our society is justified in spending more or giving greater priority to treatments that would give patients more years of life.

For example, the Pittsburgh guidance assigns a lower priority to patients anticipated to die within 1 year, or within the next 5 years if successfully treated for their acute illness (White 2020). However, it is difficult to identify a level of frailty that would generate an equivalent prediction of survival. In a meta-analysis of studies evaluating the relationship between frailty and survival, frailty was associated with an approximate 3 year difference in life expectancy for adults age 65, but a much smaller difference for older patients (>85) (Shamliyan et al. 2013). In their analysis, only frail persons over 85 had a life expectancy of less than 5 years, while only frail persons older than 100 had a life expectancy of less than 1 year!^5^ It is not clear, for example, that patients with a CFS of 5 or 6 necessarily have a short life expectancy if they are <80 years.

### Quality of Life

Third, frailty is associated with quality of life if the patient survives. That is clearly relevant to intensive care triage in terms of one metric for evaluating the effectiveness of interventions—quality adjusted life years (QALY). Cost-effectiveness analysis is used to allocate many limited resources (for example pharmaceuticals), and has been applied to intensive care (Lindemark et al. 2017). Prioritizing patients who would survive with a higher quality of life would save more QALYs than a policy that saved lives regardless of quality. Furthermore, in many countries (for example the UK), it is already common for intensive care triage to include assessment of pre-illness functional status (Bassford et al. 2019). The Clinical Frailty Scale (which is explicitly based on categories that include function and need for assistance) is closely correlated with pre-intensive care measures of activities of daily living and with cognitive decline (Guidet et al. 2020). As already noted, preexisting frailty is also associated with increases in frailty and in disability subsequent to intensive care admission, and to a lower chance of returning home. It is a predictor of long-term health-related quality of life (Bagshaw et al. 2014). However, quality of life represents a more controversial ethical criterion for ICU triage, and some pandemic guidance specifically reject this as a consideration. Twenty-seven percent of US ventilator triage guidelines reviewed in one study specifically prohibited including disability in triage decisions (Antommaria 2020).

### Age (Past Life Years)

Frailty is more common in patients of advanced age, and the degree of frailty is more common in older patients. On some views the fact that frailty is linked to older age might be an advantage for its use in rationing. That is because separate from its potential relationship with prognosis, age is potentially relevant to a different ethical consideration—fairness. Some authors have noted that it is unfair for people to die before they have had a chance to move through the usual phases of the life cycle (Emanuel et al. 2020; Persad et al. 2009). Even if their prognosis were equal, it would be arguably ethical to prioritize a young adult or a child over an older adult because the young person has not yet had a chance to live as long or to have as many positive life experiences. Studies of UK intensive care doctors indicate that they already include consideration of patient age in their admission decisions (Bassford et al. 2019). Fifty per cent of US ventilator triage policies included age (Antommaria et al. 2020).

### Length of Intensive Care Required

Finally, frailty has *not* been clearly associated with another factor for ICU triage—ICU length of stay. The duration of treatment in intensive care is relevant to triage decisions since (in the setting of resource shortage) it is related to the number of patients who are able to be treated (Savulescu et al. 2020b). Prioritizing patients who are predicted to need a shorter period of support on a ventilator means that more individual patients are able to receive support—and hence more lives can be saved. It might be expected that patients who are frail would be more difficult to wean off a ventilator and hence more likely to need a prolonged intensive care stay. In fact, the meta-analysis of ICU outcomes did not find a significantly longer length of stay in frail older patients in intensive care (Muscedere et al. 2017). That might reflect the higher mortality in this group of patients, but it does not support the inclusion of frailty in triage as a predictor of prolonged intensive care stay.

As noted, frailty was proposed more than a decade ago to have potential value in ICU triage. The evidence summarized above clearly shows that frailty is linked with three potentially highly relevant variables for triage decisions: probability of survival, longevity and quality of life (though not a fourth—ICU length of stay).

Yet, frailty has also been criticized.

## FRAILTY AND DISCRIMINATION

### Ableism

When the UK NICE guidelines were released, there was an immediate response from a number of disability advocacy groups, and some instigated a judicial review (this is a formal court based review of the lawfulness of a decision by a public body) (Hodge Jones and Allen Solicitors 2020). As noted, (Figure 2) the clinical frailty scale is increased in patients who have difficulty mobilizing and in those who need assistance in their activities of daily living. This might potentially discriminate against people with disabilities in two separate ways. First, the CFS might be used to incorrectly label patients with severe physical disabilities as frail. For example, a patient with a disabling muscle or joint condition might need support with activities of daily living. Yet they would not necessarily have the other features of the frailty syndrome (loss of biological reserve, failure of homeostasis, vulnerability to intercurrent illness). If the justification for using frailty in rationing is its relationship with probability of survival or duration of survival, it would potentially be a mistake to exclude from intensive care admission patients with stable long-standing disability. (While patients with underlying stable disability might have reduced survival post-intensive care, the relationship is likely to be less than the impact of frailty.) In response to this criticism, NICE amended its guidance to indicate that the CFS should only be used in patients over the age of 65, and should not be used for groups of patients with stable chronic disabilities (NICE 2020).

However, the modified NICE criteria remain vulnerable to a different criticism, since they still explicitly differentiate between older patients on the grounds of their degree of functional dependence. Some critics of the guidance argued that categorizing patients by care needs for critical care triage was “ableist and dangerous” (Pring 2020). Some of these criticisms are general concerns about resource shortages and rationing. For example, a group of UN special rapporteurs released a statement in late March that “Everyone, without exception, has the right to life-saving interventions and this responsibility lies with the government” (United Nations 2020). Clearly, one valuable response to the shortage of ICU beds would be to increase ICU capacity to avoid the need to choose between patients. Yet, guidance like that from NICE is designed to be used if such measures have been attempted and have failed. At that point, there is no way to treat all patients, and decisions need to be made.

Is it discrimination to use frailty in ICU triage? In one sense, all triage decisions are discriminatory—they necessarily involve “discriminating” or choosing between potential patients. The important question is whether the criteria used for such choices are ethically justified or not, whether this is “just” or “unjust” discrimination. The clinical frailty scale *does* distinguish between patients on the basis of criteria that include degrees of disability. Yet, it is not the disability per se that the score is measuring—rather it is the underlying physiological and physical vulnerability. It is this underlying vulnerability that is then associated with intensive care outcome and that justifies the ICU triage.

On the other hand, the answer to whether frailty-based triage represents disability discrimination might also depend on how much the justification depends on the relationship between frailty and post-intensive care quality of life. If quality of life is a key component of the justification it would be more difficult to defend against the ableism objection. One important question, beyond the scope of this paper, is whether disability discrimination is always necessarily problematic in rationing (Savulescu 2001). That may depend on the type and severity of the disability. For example, it would clearly be ableist to deny patients with severe dementia or severe disorders of consciousness (e.g., persistent vegetative state) medical treatment that would be given to other patients. Yet, almost all health systems would exclude such patients from receiving solid organ transplants even if they would otherwise medically be good candidates, and such discrimination is (to many people at least) unquestionably ethically justified. One way of defending such policies is to point out that resource allocation in a pluralistic society necessarily involves balancing different competing ethical values, including (but not necessarily limited to) benefit and fairness/equality.(Wilkinson and Savulescu 2018) Providing scarce resources (such as solid organs) to patients with severe disorders of consciousness would mean that we give ethical weight *only* to equality and not at all to benefit. However, plausibly, if we wish to balance these ethical values, that would mean seeking to provide equal treatment in some cases (for example where patients have lesser degrees of disability), but permitting prioritization in other, more severe cases. (Wilkinson and Savulescu 2018) Such an argument could imply that milder degrees of frailty should not be grounds for exclusion from intensive care since this would give too little weight to fairness and equality.

### Ageism

One of the motivations for NICE triage guidance was potentially to avoid age-based rationing. Yet, the guidance could be criticized along the same lines. For one thing, the modification of the guidance to only use CFS in patients over a particular age appears to have reduced concerns about disability discrimination at the cost of explicit age-based discrimination. Of course, the guidance does not exclude all patients above the age of 65 from intensive care admission—only (potentially) those who are at frail. Yet, as McNally and Lahey have noted, a policy of discrimination against a subgroup of the elderly—those who are frail and vulnerable, does not avoid concern about ageism (McNally and Lahey 2015). (Indeed, on some views, this might be seen to be doubly discriminatory—since it identifies those who have two protected characteristics—old and disabled. A policy that disadvantaged people of both a particular race and gender—would not be able to resist the claim that it was racist by insisting that it only affected a subgroup of people of that race…).

One potential justification for the revised NICE guidance is that frailty in younger patients is not necessarily associated with sufficiently poor prognosis to exclude from ICU admission. For example, in a Canadian intensive care cohort, the 1-year mortality of frail patients <65 years was 36% compared with 59% of frail patients ≥65 years) (Bagshaw et al. 2016). Indeed, that is the potential justification for the proposed (but never adopted) UK triage guidance that assigned increasing triage scores to patients with increasing age (and with increasing numbers of co-morbidities) (Figure 1).

Frailty based triage would be highly likely to disproportionately affect older patients. As with disability, the question is whether such discrimination is just or unjust. The answer to that will obviously depend on whether rationing is ethically accepted at all, and which criteria are thought to be acceptable. There is a considerable ethical literature on the question of age-based rationing. It is beyond the scope of this paper to review that in detail here, except to note that on a number of influential accounts, including the prudential lifespan approach (Daniels 2008) and capabilities approach (Jecker 2013) this is not always unjust.^6^

If it is justified to prioritize patients with the highest probability of survival, that would appear to permit frailty-based triage. What about duration of survival or longevity? Prioritizing those who would survive for more years might discriminate against the old. However, the use of frailty is relevant and valuable here. It would be unjust to automatically exclude all patients of a particular age (say 75) on the basis that they have too few remaining years of life. Seventy-five-year-olds can vary widely in their life expectancy and may indeed have a longer life expectancy than considerably younger patients. Using frailty as a proxy for life expectancy would *not* be ageist or unjustly discriminatory.^7^ It would be at most indirect discrimination, and on that basis, not necessarily problematic (Savulescu et al. 2020a).

### Frailty and Socio-Demographic Inequality

Finally, one concern about the use of frailty in triage decision-making is that its use would contribute to or reinforce socio-demographic inequality (Lewis et al. 2020).

There have been wide concerns about the impact of the COVID-19 pandemic on inequality. In some countries, rates of coronavirus infection have been higher in individuals from ethnic minorities (Martin et al. 2020) or economically disadvantaged communities (Finch and Hernández Finch 2020) and these groups also have a higher mortality rate from COVID-19 (Golestaneh et al. 2020).

Incorporating frailty into triage decisions might reinforce these worse outcomes. Frailty is not evenly distributed among the older population. Studies indicate that rates of frailty are potentially higher in some ethnic groups (Franse et al. 2018) and those of low-socio-economic status (Franse et al. 2017). So patients from these groups might be less likely to be treated in intensive care if frailty-based triage were permitted.

One potential concern arising from this association represents a general challenge to rationing and triage. If a patient or a group of patients have preexisting poor health and then that poor-health is cited as a reason to give them a lower priority for access to medical treatment (such as intensive care), there is an intuitive sense that this is unfair, and that they are “doubly disadvantaged” (Harris 1987). Such an argument might lead someone to reject incorporation of frailty in triage. However, in fact this argument would lead to rejection of all forms of triage (including those based on severity of illness, or chance of survival).

It is important to distinguish both the source of this intuitive concern and the best way of responding to it. First, it is of course regrettable that there are insufficient health care resources to provide intensive care to all who might benefit. That should lead us to look at ways to increase the availability of treatment, if possible. However, in the meantime, rationing is unavoidable. Second, social inequalities in health and life expectancy are, rightly, a serious ethical concern. We must pay attention to the social structures that generate such inequality and to ways that healthcare systems can mitigate the impact.

But, the basic aim of triage in the setting of limited resources is to distribute treatment that cannot be provided to all potential beneficiaries. It is not a tool for addressing social or health inequalities. Triage should be based on ethically salient considerations, such as those outlined above (e.g., chance of survival, longevity, duration of treatment) and not on factors that are ethically irrelevant. However, as noted above, if triage on the basis of those ethical factors indirectly disadvantages some subgroups, that is not direct discrimination and not itself unethical.

One possibility, rather than rejecting outright the use of frailty in triage decisions, would be to adjust for its distribution within socio-economic or ethnic subgroups. For example, it would be possible to use a higher frailty score as a threshold for those who are members of a particular ethnic group, or include demographic characteristics within a triage score in a way that prioritizes those from disadvantaged groups. Such an adjustment would be ethically complex to determine (which socio-demographic characteristics should be included, how much adjustment should be made to the triage score?) It would be practically complex to administer fairly (who should decide whether someone is part of a particular ethnic group, or is sociodemographically disadvantaged?) Crucially, any such social adjustments to triage scoring will necessarily result in reduced overall survival from intensive care.

This problem is not unique to intensive care rationing. Norm Daniels has noted that public health sometimes faces a conflict between two central goals—that of improving population health and reducing health inequalities (Daniels 2019). There can be reasonable disagreement about how to act when these competing priorities come into tension, and as a consequence, societies ought to adopt a fair process for deciding how to balance them (Daniels 2019). Drawing on Daniels, it would be important for there to be transparent deliberation about the approach to rationing of intensive care in the context of a pandemic including incorporating the input of members of disadvantaged groups. Of relevance, Maryland engaged in just a process of public deliberation about ventilator allocation prior to the COVID-19 pandemic (Biddison et al. 2018). It did not explicitly consider ‘frailty’ per se, but did yield a consensus in favor of prioritization on the basis of several of the factors linked to frailty including chance of survival and length of survival. (This public deliberation contributed to the aforementioned Pittsburgh COVID-19 guidance).

## HOW SHOULD FRAILTY BE USED IN RATIONING?

I have argued that it is not necessarily discriminatory to ration on the basis of frailty. States or health systems that do not currently include this factor should seriously consider incorporating it into allocation. However, there are some additional steps that are necessary to ethically include frailty in ICU triage. In this section, I will compare UK guidance with that produced at the same time in Ontario, Canada.

### Explicit Ethical Justification

Existing UK guidance documents recommending the assessment of frailty in triage do not make clear why frailty is relevant. For example, the Intensive Care Society guidance claims that their tool (incorporating age, frailty and co-morbidities) aims to identify “those patients most likely to benefit” (Intensive Care Society 2020). NICE guidance similarly refers only to ‘whether patients would benefit’ from critical care organ support (NICE 2020). Yet it is unclear from these documents how likelihood of benefit is understood, whether this refers only to chance of survival, or incorporates life years or quality of life.

Explicit justification should avoid euphemisms such as “clinically appropriate” or “ability to benefit”. This is important to ensure that resource allocation is transparent and has democratic legitimacy. It enables the wider community to understand the basis on which allocation will occur and makes clear that allocation is necessarily value-based and not (purely) a question of scientific or medical expertise. It allows allocation to be openly discussed and debated (and revised, if appropriate). It would avoid one potential concern about the use of frailty—that it represents an attempt to discriminate by stealth. Since frailty is associated with multiple different prognostic factors, politicians or policy makers might cite the less controversial associations (e.g., survival), while actually being motivated by other factors (e.g., quality of life or age). This is not to accept that those other factors are necessarily ethically problematic (see above). However, if they are to be incorporated into allocation, this should be done openly. Explicit justification is also necessary for wider application of rationing (see below).

As an example of more explicit justification, the Ontario guidance explains that the listed criteria identify patients who have “a low probability of surviving an acute illness” or “a low probability of surviving more than a few months regardless of the acute episode of critical illness” (Downar 2020). However, the Ontario guidance appear to also include (without explicit justification) quality of life in their allocation policy. For example, they exclude patients with severe baseline cognitive impairment and combine the risk of death “or poor outcome” [undefined] for patients with neurologic events (Downar 2020).

### Consistent Application

If frailty is relevant for ICU triage, it should not be assessed only in subgroups of patients. For that reason, it was potentially problematic for the NICE guidance to restrict frailty assessment to patients over the age of 65. As discussed in section III, a significant proportion of ICU patients younger than age 65 are clinically frail, and this is correlated with their outcome. It is potentially ageist to ignore frailty in the triage assessment of younger patients, but to consider it in older patients.

Consistent application does not mean that assessment needs to be identical, or that interpretation of assessment will always be identical. For example, younger frail patients have a higher chance of survival (and longer life expectancy) than older similarly frail patients. That would potentially justify a different decision—and is suggested by the draft UK national framework that incorporated both age and frailty into a combined score. Frailty in some patients (for example with stable physical disability) may need to be assessed with a different tool because of the potential confounding with the CFS. However, that does not mean that subgroups of patients should be excluded from assessment. Patients with stable disabilities may (and often will) develop superimposed frailty as they age. If their prognosis is poor, it would be wrong to admit them to intensive care in preference to a patient with better prognosis out of a desire to avoid discrimination.

The Ontario guidance includes frailty scores in their exclusion list for all patients (regardless of age) (Downar 2020). This appears to be more consistent in one sense—however, risks unjustly excluding some younger frail patients whose prognosis may be better than the frail older patients.

### Triage Equivalence

Finally, ICU triage should not be restricted to frailty. If it is justified to exclude some frail patients from ICU admission on the basis of their prognosis, it would also be justified to exclude other patients with a similar prognosis, but who are not frail. Indeed, it would be ethically inconsistent and problematic to admit those patients to intensive care. This suggests that one important step for a triage framework that includes frailty is to identify a level of prognosis equivalent to the admission threshold. We could call this “Triage equivalence.”

Triage Equivalence: Patients with equivalent relevant features should be treated similarly for triage decisions. A triage equivalent group are patients with prognosis equivalent to those patients who are currently admitted to ICU or declined admission.

This principle is one of the main reasons why it is necessary to be explicit about the ethical basis for triage decisions (above). It is only possible to apply triage equivalence if the justification for triage is made clear.

For example, NICE guidance implies that in patients with a CFS of 5 or above, the appropriateness of ICU admission should be questioned. Patients needing intensive care admission with a CFS of 5 have approximately 50% 1-year survival (Brummel et al. 2017). If the relevant understanding of ICU benefit is *chance of survival,* this suggests that other (non-frail) patients who also have a 50% chance of 1-year survival are “triage equivalent” and should also have the appropriateness of admission considered. This might have radical implications, since it would potentially exclude a number of patients with major trauma, stroke, and many patients post cardiac arrest. However, if the relevant metric is probability *and* duration of survival (life years), or probability, duration and quality of survival (QALY), that would potentially mean a different equivalent population.^8^

The Ontario guidance endorses exactly this notion of triage equivalence by identifying a set of conditions with equivalent prognosis. For example, it includes a list of conditions with estimated 50% mortality, equivalent to a frailty score of ≥5 (Downar 2020). (The guideline includes three different thresholds depending on the level of resource shortage, corresponding to 30%, 50% and 80% mortality). However, as already noted, the guideline is somewhat ambiguous about outcome since it implicitly, (but not explicitly) includes within prognosis equivalence predicted short duration of survival and reduced quality of life.

One advantage of the concept of triage equivalence is that it allows triage thresholds to be revised in response to changes in ICU availability and to changes in data about outcome. For example, if data were published indicating that patients with a CFS of 5 had a better than expected outcome following COVID, that would potentially support a shift of the triage threshold to reflect a prognosis equivalent to that originally expected. (For example, it might mean moving the bar up to a CFS of 6).

The published Intensive Care Society guidance does not indicate a threshold for ICU triage. That had potentially been seen as too controversial a feature of the draft guidance. It is not possible, using the ICS guidance, to assess which combination of age, comorbidity and frailty would justify a particular triage decision. That is potentially a serious flaw with that guidance and makes it impossible either to apply the guidance consistently or to assess for triage equivalence.

## CONCLUSIONS

In many respects, frailty might be almost a perfect ICU rationing criterion—potentially attractive according to multiple different theories of rationing, and in combination identifying patients who are less likely to survive, likely to survive for a shorter period, and with greater functional impairment (as well as having potentially already experienced more life).

Frailty is ethical to use in ICU triage. It has been studied prospectively in multiple studies in different countries and is independently associated with intensive care outcome. It is able to be rapidly assessed in a way that appears to be feasible, on the basis of information available at the time of presenting to hospital (Pugh et al. 2018).^9^ It is not, contrary to the views of critics, unjustly discriminatory to draw on frailty in ICU triage.

Frailty has several distinct advantages over the other much-criticized “F” word in medical ethics—“Futility”. Frailty is conceptually clear and measurable. It can be quantified. It is considerably less subjective than a judgment that treatment is “futile”. It is very clear, when frailty is incorporated into pandemic guidance that the basis for decisions is that of limited resources, and not that of the best interests of the patient (Wilkinson et al. 2018b).

However, I have suggested three important steps to improve the ethical incorporation of frailty into triage decisions. Triage criteria (i.e., frailty) should be assessed consistently in all patients referred to the intensive care unit. Guidelines must make explicit the ethical basis for the triage decision. Finally, this should then be applied, using the concept of triage equivalence, to allow ethically consistent triage applicable to all patients and age groups needing critical care.

## Footnotes

1 One question is why patients who are terminally ill (but do not have other features of frailty) are included within the frailty scoring system. It might be that terminal illness is associated with physiological vulnerability in a way that is not captured by the clinical frailty tool. Alternatively, this might be understood as a way to include other patients with short survival within the ambit of a “frailty” assessment. It would arguably be conceptually clearer to include such patients using Triage Equivalence (see below). Furthermore, it is not clear that dementia is necessarily equivalent to frailty. A younger patient with moderate dementia might not have underlying frailty, though they would be in need of support with tasks of daily living.

2 Even in this setting, there might be concerns about what information is judged to be accurate and relevant and provided to patients or surrogates. One challenge is that existing information (for example about survival) may be influenced by treatment decisions and consequently be affected by self-fulfilling prophecies (Wilkinson 2009). For example, in a number of the studies examining intensive care outcome, prior frailty was associated with treatment withdrawal and withholding decisions (Guidet et al. 2018). This makes it difficult to provide information to patients about what the outcome would be if treatment were provided.

3 Official notes from a meeting of a national ethical advisory group, the UK Moral and Ethical Advisory Group 25th March indicate that this draft document was actively debated at the meeting. An explanatory note was added to the record of the meeting to indicate that the guideline “was not progressed any further as it became clear it was not needed.” The note also documented that the group’s discussion had contributed to the development of the later Intensive Care Society guidance. Official note. Moral and Ethical Advisory Group (MEAG) – meeting 4. 25 March 2020. https://www.gov.uk/government/groups/moral-and-ethical-advisory-group. Accessed June 16, 2020.

4 While the use of illness severity scores in triage algorithms are widespread, they are not without criticism. For example, some have noted that SOFA scores in patients with COVID-19 might be disproportionately higher in certain racial or ethnic groups (Galiatsatos et al. 2020). I will consider below the objection that the use of frailty in triage would reinforce racial or other social inequality. Some recent triage guidelines have moved away from illness severity scores, as they do not appear to predict well patients with low probability of surviving (Downar 2020).

5 The meta-analysis calculated life expectancy for frail compared with non-frail patients but did not analyse degree of frailty or generate estimates for those who are severely frail. It was focused on mortality for community dwelling adults (so may not be directly applicable to patients needing intensive care admission).

6 Jecker (2013) argues that the capabilities approach is preferable to Daniel’s prudential lifespan account, in that it respects the equal worth of all human beings and safeguards their basic capabilities. She does not specifically consider resource allocation during a pandemic, but argues that the Capabilities approach could justify rationing publicly funded life extending care for the elderly in order to provide it to the young on the basis of society’s duty to protect and secure our ability to secure a normal life span.

7 If the association between age and past life years is providing a significant justification (association with age and past life years) is providing a significant justification for the use of frailty in triage, that would represent direct discrimination. (Though on some views this would still be justified).

8 It is not possible from existing published date to evaluate how many life years would be saved by admitting patients to intensive care with a CFS of 5. This is likely to vary significantly with age. Similarly, the number of QALYS saved is not clear. However, that suggests (if these are the relevant ethical considerations) there is a need for better data on the benefit of intensive care admission in order to inform triage decision-making.

9 Pugh’s systematic review was unable to find sufficient evidence on the reliability of the CFS, though this appeared to be the most widespread tool for assessing frailty.
